# Long non-coding RNA metastasis-related lung adenocarcinoma transcript 1 (MALAT1) forms a negative feedback loop with long non-coding RNA colorectal neoplasia differentially expressed (CRNDE) in sepsis to regulate lung cell apoptosis

**DOI:** 10.1080/21655979.2021.2023727

**Published:** 2022-03-18

**Authors:** Caifang Yue, Muhan He, Yanping Teng, Xiaoli Bian

**Affiliations:** Department of Critical Care Medicine, No. 1 Hospital Attached to Jiamusi University, Jiamusi City, P. R. China

**Keywords:** MALAT1, CRNDE, human bronchial epithelial cells, apoptosis

## Abstract

It has been reported that long non-coding RNAs (lncRNAs) metastasis-related lung adenocarcinoma transcript 1 (MALAT1) and colorectal neoplasia differentially expressed (CRNDE) play opposite roles in sepsis. Therefore, we explored their potential interaction with sepsis. To this end, we determined MALAT1 and CRNDE levels using RT-qPCR in plasma samples collected from healthy controls (n = 60) and sepsis patients (n = 60) before and after treatment and the effects of MALAT1 and CRNDE overexpression in human bronchial epithelial cells (HBEpCs) on the expression of each other and HBEpC apoptosis. RT-qPCR analyses showed that MALAT1 was upregulated, while CRNDE was downregulated in sepsis and overexpression of MALAT1 and CRNDE downregulated the expression of each other. After proper treatment, MALAT1 was downregulated and CRNDE was upregulated in sepsis. Lipopolysaccharides (LPS) treatment of HBEpCs upregulated MALAT1 and downregulated CRNDE. Cell apoptosis analysis showed that MALAT1 overexpression promoted, while CRNDE overexpression inhibited LPS-induced HBEpC apoptosis. Moreover, CRNDE overexpression attenuated the effects of MALAT1 overexpression. Overall, MALAT1 might form a negative feedback loop with CRNDE in sepsis to regulate lung cell apoptosis.

## Introduction

Sepsis is a severe clinical condition due to body’s extreme systemic immune response to its own organs and tissues [1]. The most common cause of sepsis is bacterial infections [2]. The development of sepsis and septic shock will result in the failure of multiple organs, leading to an unacceptably high mortality rate [3]. In the United States, at least 1.7 million adults are diagnosed with sepsis, causing about 270,000 deaths every year [4]. It is estimated that even after effective treatments, such as the use of antibiotics, oxygen, and intravenous fluids, more than 50% of patients with septic shock or severe sepsis will die of this disease [5]. Therefore, novel therapeutic approaches are needed to improve the survival of sepsis patients.

Infections are not sufficient for the development of sepsis [6]. More and more studies have elucidated that multiple molecular pathways are involved in the initiation and progression of sepsis and sepsis-related organ failures [7,8]. Increased understanding of the molecular mechanisms of sepsis may provide novel targets for the treatment and prevention of sepsis-related organ dysfunctions by regulating the expression of critical genes [9]. Long non-coding RNAs (lncRNAs) have no protein-coding capacity, but they could regulate the expression of their target genes to be critical players in human diseases, such as sepsis [10,11]. Therefore, lncRNAs may serve as potential targets for sepsis treatment. However, the functions of most lncRNAs remain unclear. LPS-induced inflammatory response plays a critical role in sepsis [12]. It has been reported that lncRNAs Metastasis Associated Lung Adenocarcinoma Transcript 1 (MALAT1) and Colorectal Neoplasia Differentially Expressed (CRNDE) have opposite roles in LPS-induced organ damages [13,14], indicating the potential interaction between them. Therefore, we hypothesized that MALAT1 might interact with CRNDE to participate in LPS pathways involved in sepsis. Therefore, this study was carried out to analyze the roles of MALAT1 and CRNDE in sepsis and to explore their potential interactions.

## Materials and methods

### Sepsis patients and healthy controls

The study included both sepsis patients (n = 60, 34 males and 26 females; 38–62 years; 49.8 ± 5.6 years) and healthy controls (n = 60, 34 males and 26 females; 38–62 years; 49.9 ± 5.7 years) who enrolled at No. 1 Hospital Attached to Jiamusi University from May 2017 to May 2019. All sepsis patients were diagnosed for the first time. The main cause of sepsis was bacterial infections. All patients survived for more than 3 months after admission. In view of the fact that other clinical disorders or treatments may also affect the expression of certain genes, this study excluded patients with initiated therapy or complicated with other clinical disorders (not the co-morbidities of sepsis, such as cancers and chronic diseases). The healthy controls were subjected to systemic physiological tests and had normal physiological functions. All participants signed informed consent.

### Plasma and treatment

Fasting blood (3 ml) was extracted from all patients before therapy and healthy individuals and centrifuged at 1200 g for 15 min to collect plasma samples. All patients were treated with antibiotics in combination with oxygen and intravenous fluids. After 3 months of treatment, fasting blood was extracted again from each patient to prepare plasma samples. RNAs were extracted from plasma samples immediately after plasma preparation.

### Human bronchial epithelial cells (HBEpCs)

With lung injury in sepsis as a focus, HBEpCs (Sigma-Aldrich) were used as the cell model. HBEpCs were cultured in Bronchial Epithelial Cell Medium (Cat. #3211; PromoCell) at 37°C in a humidified incubator with 5% CO_2_. At passages 3 to 5, cells were harvested and used for subsequent experiments. To explore the effects of LPS on gene expression, HBEpCs were treated with LPS at doses of 0, 1, 2, 5, and 10 µg/ml for 48 h before use.

### Cell transfections

MALAT1 or CRNDE expression vector was constructed with pcDNA3.1 as the backbone vector (Invitrogen). MALAT1 or CRNDE expression vector (1 μg) or negative control (NC) vector (1 μg) was transfected into 10^8^ HBEpCs using lipofectamine 2000 (Invitrogen). After transfections, cells were cultured for 48 h before use. Cells without transfections were used as control (C).

### RNA preparations

Total RNAs were isolated from both plasma samples and HBEpCs using Ribozol reagent (Invitrogen) and treated with gDNA eraser for 2 h at 37°C to remove genomic DNAs. RNA integrity was tested using 5% Urea-PAGE gel. OD values at 260 nm and 280 nm were measured, and their ratio was calculated.

### RT-qPCRs

Total RNA samples with a 260/280 ratio close to 2.0 (pure RNA) were used as templates to synthesize cDNAs through reverse transcriptions, which were performed using SSRT IV system (Invitrogen). With cDNA samples as templates, qPCRs were performed using SensiFAST™ Real-Time PCR Kit (Bioline) with 18S rRNA as the internal control to normalize the expression levels of MALAT1 and CRNDE. Each experiment included three technical replicates, and gene expression levels were normalized using the 2^−ΔΔCt^ method.

### Cell apoptosis assay

To induce cell apoptosis, HBEpCs with transfection were cultured in 6-well plates with 8000 cells in a 2 ml medium supplemented with 0 (baseline control) or 10 µg/ml LPS per well for 48 h. After that, cells were washed with ice-cold PBS, stained using PI and FITC-annexin V (Sigma-Aldrich) in the dark for 20 min, and subjected to flow cytometry to separate apoptotic cells. Each experiment was performed on three replicates.

### Statistical analysis

Gene expression levels in plasma samples were expressed as mean ± standard deviation (SD) of 3 independent values. Differences between two groups and two time points of the same group were analyzed by unpaired t test. Differences among multiple groups were analyzed by ANOVA Tukey’s test. Correlation analyses were performed using linear regression. P < 0.05 was set to be statistically significant.

## Results

### Altered MALAT1 and CRNDE expression levels were observed in sepsis

**Figure 1.**
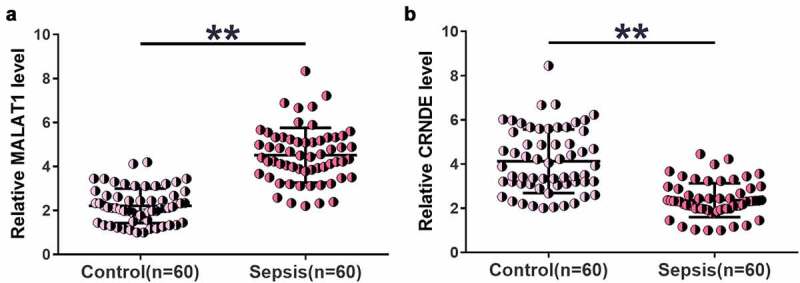
Altered MALAT1 and CRNDE expression levels were observed in sepsis.

We first examined changes in MALAT1 and CRNDE levels in plasma samples from both sepsis patients (n = 60) and healthy controls (n = 60) using RT-qPCR. Higher MALAT1 (Figure 1(a), p < 0.01) and lower CRNDE (Figure 1(b), p < 0.01) levels were identified in sepsis samples than in control samples. Therefore, altered expression of MALAT1 and CRNDE may participate in sepsis.

MALAT1 (a) and CRNDE (B) levels in plasma samples from both sepsis patients (n = 60) and healthy controls (n = 60) were determined by RT-qPCR. Data are presented as the average of three technical replicates. **, p < 0.01.

### MALAT1 was downregulated and CRNDE was upregulated after treatment

**Figure 2.**
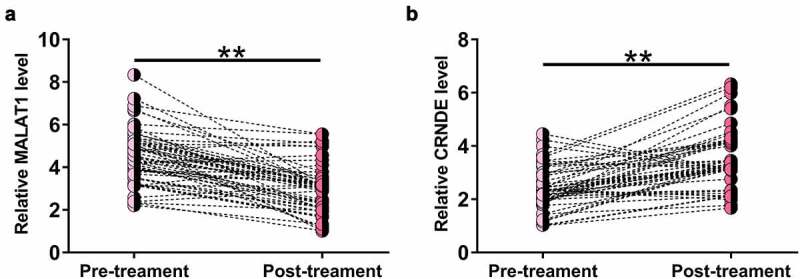
MALAT1 was downregulated and CRNDE was upregulated after treatment.

To determine the effects of treatment on MALAT1 and CRNDE expression, MALAT1 and CRNDE expression levels in plasma samples from sepsis patients at 3 months after treatment were also determined by RT-qPCR. Compared to pretreatment levels, significantly lower MALAT1 (Figure 2(a), p < 0.05) and higher CRNDE (Figure 2(b), p < 0.05) levels were observed after treatment, indicating that MALAT1 and CRNDE expression may respond to treatment. It is worth noting that after treatment, the relative MALAT1 level was 3.78 ± 1.75, still significantly higher than that of 2.14 ± 1.11 in the controls (p < 0.01). In contrast, the relative CRNDE level after treatment was 4.01 ± 2.12, which was close to that of 3.98 ± 1.17 in the controls (p > 0.05).

MALAT1 and CRNDE expression levels in plasma samples from sepsis patients at 3 months after treatment were determined by RT-qPCR. Shown are the pre- and post-treatment levels of MALAT1 (a) and CRNDE (b). **, p < 0.01.

### MALAT1 and CRNDE negatively regulated each other in HBEpCs

**Figure 3.**
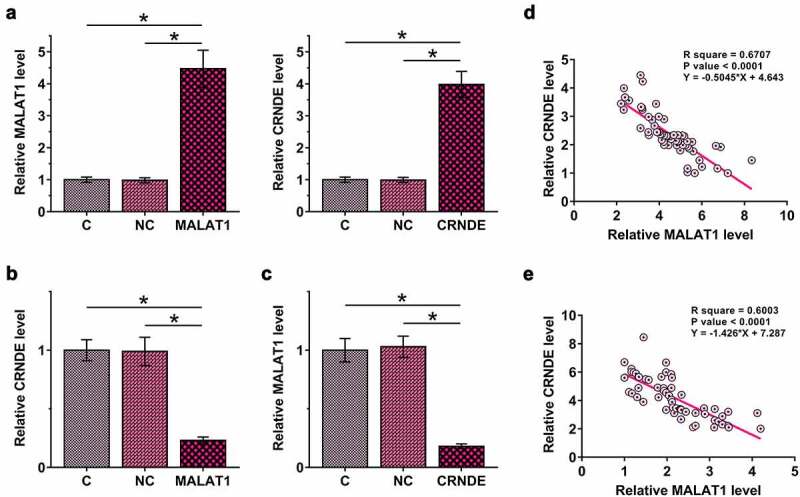
MALAT1 and CRNDE negatively regulated each other in HBEpCs.

To study the interaction between MALAT1 and CRNDE, MALAT1 or CRNDE expression vector was transfected into HBEpCs, and the transfections were confirmed by RT-qPCR at 48 h post-transfection (Figure 3(a), p < 0.05). It was observed that MALAT1 overexpression downregulated CRNDE (Figure 3(b), p < 0.05). In addition, CRNDE overexpression also downregulated MALAT1 (Figure 3(c), p < 0.05). Therefore, MALAT1 and CRNDE may form a negative regulation loop in HBEpCs. Correlations between MALAT1 and CRNDE across plasma samples from both sepsis patients (Figure 3(d)) and healthy controls (Figure 3(e)) were analyzed using linear regression. It was observed that MALAT1 and CRNDE were inversely and significantly correlated with each other across both sepsis and control plasma samples. Therefore, MALAT1 and CRNDE may also interact with each other in the human body.

MALAT1 or CRNDE expression vector was transfected into HBEpCs, and the transfection was confirmed by RT-qPCR at 48 h post-transfection (a). The effects of MALAT1 overexpression on CRNDE (b) and the effects of CRNDE overexpression on MALAT1 (c) were explored using RT-qPCR. Correlations between MALAT1 and CRNDE across plasma samples from both sepsis patients (d) and healthy controls (e) were analyzed using linear regression. Data are shown as the mean ± SD values of three independent replicates. C, control cells without transfection, NC, negative control cells transfected with empty vector. *, p < 0.05.

### MALAT1 and CRNDE interacted with each other to regulate LPS-induced HBEpC apoptosis

**Figure 4.**
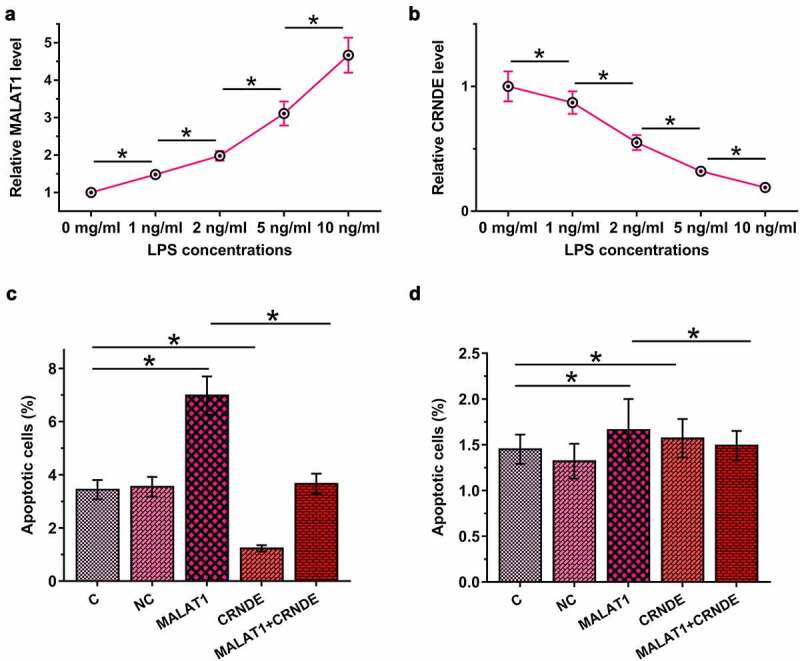
MALAT1 and CRNDE interacted with each other to regulate LPS-induced HBEpC apoptosis.

Sepsis is closely associated with LPS-induced cell apoptosis. To this end, HBEpCs were cultured in media supplemented with 0, 1, 2, 5, and 10 µg/ml LPS for 48 h, and MALAT1 and CRNDE levels were determined by RT-qPCR. LPS treatment upregulated MALAT1 (Figure 4(a), p < 0.05) and downregulated CRNDE (Figure 4(b), p < 0.05) in a dose-dependent manner. Cell apoptosis analysis showed that MALAT1 overexpression promoted LPS-induced, while CRNDE overexpression inhibited HBEpC apoptosis. Moreover, CRNDE overexpression attenuated the effects of MALAT1 overexpression (Figure 4(c), p < 0.05). Interestingly, without LPS treatment, MALAT1 and CRNDE failed to significantly affect HBEpC apoptosis (Figure 4(d)), suggesting that the roles of MALAT1 and CRNDE in the apoptosis of human bronchial epithelial cells are mediated by LPS.

HBEpCs were cultured in media supplemented with LPS at doses of 0, 1, 2, 5, and 10 µg/ml for 48 h, and the expression levels of MALAT1 (a) and CRNDE (b) were determined by RT-qPCR. Cell apoptosis assay was performed to analyze the roles of MALAT1 and CRNDE in regulating LPS-induced HBEpC apoptosis (c). The participation of MALAT1 and CRNDE in non-LPS-induced HBEpC apoptosis was explored using cell apoptosis assay (d). Shown are the mean percentages ±SD values of cells positive to both PI/FITC and to PI only from three independent replicates. C, control cells without transfection; NC, negative control cells transfected with empty vector; MALAT1+ CRNDE, cells overexpressed with both MALAT1 and CRNDE. *, p < 0.05.

## Discussion

This study explored the interaction between MALAT1 and CRNDE in sepsis. We found that MALAT1 was upregulated and CRNDE was downregulated in sepsis. In addition, MALAT1 and CRNDE might form a negative regulation loop to participate in LPS-induced HBEpC apoptosis.

MALAT1 is a well-characterized lncRNA in cancer biology [15]. It is upregulated in many cancers and promotes cancer development mainly by regulating cancer-related gene expression [15]. Yong et al. recently reported that MALAT1 is upregulated in a mouse sepsis model and enhances skeletal muscle cell apoptosis by downregulating BRCA1 to promote disease progression [13]. Consistently, we observed MALAT1 upregulation in patients with sepsis. Interestingly, MALAT1 overexpression promoted LPS-induced HBEpC apoptosis. Therefore, MALAT1 may regulate the apoptosis of multiple kinds of cells induced by LPS to participate in the injuries of multiple organs. In addition, MALAT1 can promote cancer development by suppressing cell apoptosis. Therefore, MALAT1 may play an opposite role in cell apoptosis in different cancers.

LncRNA CRNDE is also a characterized oncogenic lncRNA with multiple functions in cancer biology [16,17]. Wang et al. reported [18] ****that CRNDE was downregulated in a mouse sepsis model to suppress cell apoptosis via upregulating miR-181a-5p [14]. Our study also observed CRNDE downregulation in patients with sepsis and the inhibitory effects of CRNDE on LPS-induced HBEpC apoptosis. Interestingly, LPS treatment regulated both MALAT1 and CRNDE in a dose-dependent manner. Therefore, MALAT1 and CRNDE may participate in sepsis in a LPS-dependent manner.

LncRNAs participate in both physiological and pathological processes mainly by regulating the expression of protein-coding genes or other non-coding RNAs, such as miRNAs [17]. However, the interactions between different lncRNAs remain unclear. This study showed that MALAT1 and CRNDE could form a negative feedback regulation loop to participate in the LPS-induced HBEpC apoptosis. Therefore, this study enriched our understanding of the functions of lncRNAs. Our future studies will explore the underlying mechanisms. During the treatment, MALAT1 showed a delayed recovery. Therefore, besides CRNDE, other factors may also regulate MALAT1 expression in sepsis. MALAT1 and/or CRNDE only affected apoptosis of cells treated with LPS. We showed that LPS could regulate MALAT1 and CRNDE expression. Therefore, LPS may affect both cell apoptosis and MALAT1 and CRNDE expressions. Other LPS-induced factors may influence MALAT1 and CRNDE expression to affect cell apoptosis.

Although many studies have shown the involvement of lncRNAs in sepsis [19,20], the roles of most lncRNAs in this disease remain unclear and need to be further explored.

## Conclusion

MALAT1 is upregulated in sepsis and CRNDE is downregulated in sepsis. In addition, MALAT1 and CRNDE may negatively regulate each other to regulate LPS-induced HBEpC apoptosis.

## Data Availability

The datasets used analyzed during the current study are available from the corresponding author upon reasonable request.

## Highlights

1. MALAT1 was upregulated, while CRNDE was downregulated in sepsis.
2. MALAT1 overexpression promoted, while CRNDE overexpression inhibited LPS-induced lung cells the apoptosis.
3. MALAT1 might form a negative feedback loop with CRNDE in sepsis to regulate lung cell apoptosis.
